# Adverse drug reaction management in hospital settings: review on practice variations, quality indicators and education focus

**DOI:** 10.1007/s00228-022-03287-1

**Published:** 2022-02-16

**Authors:** Ar Kar Aung, Steven Walker, Yin Li Khu, Mei Jie Tang, Jennifer I. Lee, Linda Velta Graudins

**Affiliations:** 1 Department of General Medicine, Alfred Hospital, Melbourne, Australia; 2 School of Public Health and Preventive Medicine, Monash University, Melbourne, Australia; 3 Department of Pharmacy, Alfred Hospital, Melbourne, Australia; 4 Department of Medicine, NewYork-Presbyterian Hospital/Weill Cornell Medicine, New York, USA; 5 Quality Improvement Academy, Weill Cornell Medicine, New York, USA

**Keywords:** Adverse drug reactions, Reporting, Management, Quality, Practice variations, Education strategies

## Abstract

**Purpose:**

Adverse drug reactions (ADRs) contribute significantly to healthcare burden. However, they are largely preventable through appropriate management processes. This narrative review aims to identify the quality indicators that should be considered for routine monitoring of processes within hospital ADR management systems. It also examines the potential reasons behind variation in ADR management practices amongst HCPs, and explores possible solutions, focusing on targeted education programmes, to improve both the quality and quantity indicators of ADR management processes.

**Methods:**

A comprehensive literature review was conducted to explore relevant themes and topics concerning ADR management, quality indicators and educational interventions.

**Results:**

Substantial variability exists in ADR management amongst healthcare professionals (HCPs) with regard to reporting rates, characteristics of ADRs reported, quality of assessment, completeness of reports and, most importantly, risk communication practices. These variable practices not only threaten patient safety but also undermine pharmacovigilance processes. To date, quality indicators to monitor ADR management practices within hospital settings remain ill-defined. Furthermore, evidence behind effective interventions, especially in the form of targeted education strategies, to improve the quality of ADR management remains limited.

**Conclusions:**

The focus of ADR management in hospitals should be to promote patient safety through comprehensive assessment, risk communication and safe prescribing. There is a need to develop a system to define, measure and monitor the quality of ADR management. Educational strategies may help improve the quality of ADR management processes.

## Introduction

Adverse drug reactions (ADRs) are defined as a ‘response to a medicinal product which is noxious and unintended, which occurs at doses normally used in man’ and specifically denote reactions caused by the pharmacological action of the medication itself, rather than as a result of incorrect use [1]. ADRs are commonly categorised by Rawlins’ classification system into type A or type B reactions, and less commonly to other types, according to underlying pathogenesis and clinical attributes (Table 1) [2]. Preventable ADRs are those that are caused by administration errors, such as incorrect medication/dose/timing, prescription to a patient with a known allergy and inadequate monitoring [3]. Pharmacovigilance is an overarching term in ADR terminology, which encompasses safety activities and systems related to the detection, assessment, understanding and prevention of adverse effects or medication-related problems. Pharmacovigilance integrates identification of ADRs, data collection and analysis and regulatory activities as an integral part of any public health programme [4].

**Table 1:** Rawlins’ classification system of adverse drug reactions [23]

**Type**	**Underlying pathophysiology and characteristics**	**Examples**
A	Augmented pharmacological effects through PK and PD, predictable	Hypotension from calcium channel blockers
B	Bizarre effects, frequently due to immune-mediated mechanisms, not related to PK/PD properties, unpredictable	Anaphylaxis to penicillins Toxic epidermal necrolysis to carbamazepine
C	Long term effects related to dose and duration of administration	Adrenal suppression from prolonged prednisolone use
D	Long term effects resulting in carcinogenesis, teratogenesis or chronic organ damage	Cyclophosphamide and bladder cancer Amiodarone and pulmonary fibrosis
E	Effects related to withdrawal	Opiate withdrawal
F	Therapeutic effects or failure due to drug interactions	Bone marrow suppression related to concurrent methotrexate and trimethoprim/sulfamethoxazole administration

*PD* pharmacodynamics, *PK* pharmacokinetics

ADRs frequently occur in healthcare settings, contributing 3–5% of hospital admissions in both adult and paediatric populations [5–9]. Approximately 10–17% of hospitalised patients experience an ADR [6, 10]. An episode of ADR-related hospitalisation in itself is a risk factor for repeat hospitalisation due to another ADR [11]. ADR–affected patients carry a mortality risk of 10.7%, nearly 3-fold higher than those unaffected. A two-fold increase in length of hospital stay of up to 20 days can also result from ADR episodes [12]. In severe or life-threatening reactions, patients may suffer from substantial physical and psychological health impacts long-term [13, 14]. Overall, ADRs contribute in excess of 30 billion dollars to healthcare costs in the United States annually [8]. In Australia, the annual cost of medication-related admissions has also been estimated at AU$ 1.2 billion, yet 45% of ADR-related hospitalisations may be prevented using appropriate measures [9, 15].

In various healthcare settings, including hospitals, ADR episodes are assessed and reported to pharmacovigilance authorities by healthcare professionals (HCPs) including medical doctors, pharmacists and nurses [16, 17]. The reporting practices are usually voluntary, also termed ‘spontaneous reporting’. This approach has been shown to be a cost-effective method to detect new and important ADR signals [18].

Many guidelines exist to increase awareness amongst HCPs of the burden of ADRs and highlight their moral and professional obligation to report ADR episodes, so as to reduce medication-related mortality and morbidity. One such document—the World Health Organisation’s ‘Safety of Medicines’ guide—provides generic recommendations for monitoring, assessment, reporting and education of ADRs, applicable to all healthcare settings in all countries [5]. Specific national guidelines also exist to guide pharmacovigilance activities in various healthcare settings. In Australia, the Therapeutics Goods Administration (TGA) requires reporting of all suspected adverse events to new therapeutic goods, all suspected medicine/vaccine interactions, unexpected adverse events to known medications that have not been previously described or serious adverse events defined as those resulting in death or disability [19].

Despite these well-intended guidelines, several aspects of ADR management remain heavily reliant on human factors, resulting in large variations in clinical practices. HCPs’ attitudes have been known to influence suboptimal reporting rates [20]. The types of ADR reported and accuracy and completeness of reports are also known to vary depending on HCPs’ experience and settings [21]. The process of ADR evaluation requires certain technical skills, such as basic understanding of pharmacologic and immunologic principles, comprehensive history taking, accurate assessment of medication exposure timelines, diagnostic investigations and consideration of alternative causes [5]. Where an ADR occurs in complex clinical settings, inaccurate assessment and reporting can cause harm through not recognising the culprit medication, whilst implication of wrong or by-stander medications may lead to unnecessary avoidance. Inaccurate evaluation also impacts post-marketing pharmacovigilance and undermines consumer confidence in medication safety.

Therefore, within each hospital, it is important to establish an ADR management system and then to regularly monitor indicators that enable benchmarking. Maintaining the quality of ADR processes reduces variations in practice and may ultimately improve patient safety. Possible indicators include monitoring of ADR reporting rates and characteristics of medications and reactions, as well as ensuring that reports meet a certain standard. In reality, although the quantitative aspects of ADR reporting can be easily evaluated, quality parameters are harder to define or measure. The quality of an ADR report is a good reflection of HCPs’ skills in assessing an ADR episode, which includes a minimum dataset of clinical information to enable evaluation of causality association between a medication and a reaction [22]. A quality report is largely influenced by technical ability, knowledge and experience, coupled with the awareness of imprecise science behind causality assessment. Furthermore, effective documentation and communication of risks to patients and other HCPs regarding an ADR episode also constitute an important quality measure.

In the context above, this narrative review attempts to highlight the key processes and outcomes that should be routinely considered as quality indicators in ADR management systems, specifically in hospital settings, by exploring the current literature on ADR assessment and reporting practices. It also provides an understanding of potential reasons for sources of variation in ADR management. Furthermore, it explores possible solutions, focusing on targeted education programmes, to improve both the quantity and quality indicators of ADR management processes.

## Variations in ADR management

### ADR reporting rates

Despite the cost-effectiveness of spontaneous reporting, under-reporting of ADRs by HCPs remains a global issue. Following the introduction of a spontaneous ADR reporting system in the UK in the early 1990s, it was estimated that only 10% or less of all ADRs were formally reported [23]. Moreover, a systematic review by Hazell et al. confirmed under-reporting rates to be as high as 94% with spontaneous reporting practices, when compared to the actual occurrence rates of known, suspected or expected ADR as determined through hospital or general practice-based monitoring systems [24]. Under-reporting rates in excess of 95% also appeared to be similar between hospital and community settings [24].

Concerningly, the reporting rate for severe or life-threatening hypersensitivity reactions also remains low. A Canadian study found that out of a total 674 episodes of toxic epidermal necrolysis (TEN) cases nationally over a 6-year period, only 4% were reported to the Canadian Adverse Drug Reaction Monitoring Program [25]. In an Australian context, a single-centre study of 81 patients with severe cutaneous adverse drug reactions found that only 71.6% of cases had ADR reports submitted [26]. Amongst 80 patients with anaphylaxis reactions to non-antimicrobial medications, a large proportion of which were due to neuromuscular blocking agents in the perioperative setting; less than half (47.5%) of the cases were reported for further evaluation [27]. Similarly, in a multicentre Australian study concerning antimicrobial-related anaphylaxis episodes, only 43.7% of 222 episodes were reported to ADR management systems within respective hospitals [28].

### Characteristics of ADR reports

The type and characteristics of ADRs reported are also known to differ between professions, clinical expertise, practice settings, medication types and country of origin.

For instance, a Norwegian study found that pharmacists were more likely to report ADRs related to cardiovascular, gastrointestinal, metabolic and respiratory systems, whilst physicians were more likely to report ADRs related to musculoskeletal, anti-neoplastic and immunomodulating medications [17]. A similar study in the Netherlands, predominantly involving community pharmacists, found that pharmacists reported ADRs related to skin and eyes, whereas physicians tended to report ADRs related to cardiovascular system, liver dysfunction and psychiatric disorders [16]. Such reporting biases were likely influenced by the clinicians’ expertise, types of patients seen and ADR manifestations encountered in different clinical settings. Additionally, differences in ADR definitions, reporting requirements, thresholds and target medications (e.g. biologics) to be reported, as determined by national pharmacovigilance authorities, may also contribute significantly to variations in certain types of ADRs preferentially reported by HCPs.

In terms of the nature of reactions, an Australian study found that of 555 ADR reactions over a 2-year period, HCPs preferentially reported a higher number of immunologically mediated (Type B) reactions than pharmacologically related Type A reactions (73.7% vs. 23.1%), perhaps reflecting a biased perception that Type B reactions were more harmful to patients [21]. The majority of reactions reported (> 70%) were of at least moderate severity. The time to assessment and reporting of severe cutaneous adverse reactions (i.e. drug rash/reaction with eosinophilia and systemic symptoms (DRESS), Stevens-Johnson syndrome/toxic epidermal necrolysis (SJS/TEN), acute generalised exanthematous pustulosis (AGEP)) was also longer compared to other reaction types, with a median reporting time of 12 days from onset of symptoms (IQR 5–18.5), further highlighting the intrinsic challenges and complexities associated with the assessment of these severe reactions.

### Quality of ADR reports

The quality and completeness of an ADR report refer to the minimum dataset required to determine causality [22]. Concise and accurate information regarding an ADR episode, including relevant clinical narrative and medications timeline, is helpful in identifying culprit medications and verifying the strength of causality. A careful and meticulous causality assessment is of utmost importance where multiple medications are involved. Tools such as Adverse Drug Reaction Probability Scale (also known as Naranjo algorithm) [29], Algorithm for Assessment of Drug Causality in Stevens-Johnson Syndrome and Toxic Epidermal Necrolysis (ALDEN) [30] and the Registry of Severe Cutaneous Adverse Reaction (Regi-SCAR) criteria [31] may also aid in clarifying the strength of causality in certain ADR conditions. However, these causality assessment tools have significant limitations, and new tools are being developed to provide better sensitivity and specificity in evaluation [32].

Data requirements for ADR reports are not uniform across the board. A multinational study by Bandekar et al. noted concerning variations and discrepancies in reporting forms between countries, with incomplete information, which may potentially lead to delayed recognition and withdrawal of medicines with unacceptable safety profiles by the regulatory authorities in the post-marketing phase [33]. The authors suggested that, ultimately, standardised ADR reporting methods across the board would provide a more complete, comparable and reliable dataset to allow timely recognition of important ADR signals [33, 34].

To assess the completeness of an ADR report, the Uppsala Monitoring Centre developed ‘vigiGrade score’. This uses weighted input scores to assess completeness of information in the following domains: medication, dose, time-to-onset of ADR from medication initiation, indications for treatment, outcome of ADR, patient’s gender and age, country of origin, occupation of reporter, type of report (spontaneous vs. clinical study) and free-text comments [22]. A weighted penalty score is applied to missing or incomplete information dimension. Although the vigiGrade score provides a validated method to evaluate the completeness of ADR reports, its complexity is a limitation to practical application outside a research setting. Furthermore, as the vigiGrade score focuses on data dimensions, rather than individual data elements, certain aspects of the score remain counter-intuitive in determining causality (for instance, no penalty score is applied to an implicated medication being commenced after the onset of a reaction).

Amongst those that utilised the vigiGrade score to evaluate completeness, the Catalan Center of Pharmacovigilance in Spain found that of 824 reports describing serious ADRs, the rate of incomplete information ranged from 7.9 to 50.1%, with the information regarding ‘time-to-onset’ commonly missing in 17.5% [35]. Similarly, another Spanish study showed overall completion rate of 88% on the vigiGrade score for spontaneous ADR reports [36]. A Japanese study, which evaluated over 8000 reports from medical institutions, found that 75.6% were ‘well documented’ on the vigiGrade criteria, but ‘time-to-onset’ of reactions could not be determined in 14.9% of cases [37].

In studies using alternative systems of assessment, varying degrees of completeness and quality of information were evident. A Chinese study requiring 65 data items in their ADR reports found that only 10.18% of 3429 reports submitted to a regional pharmacovigilance centre were considered high quality [38]. Another study from a western Chinese pharmacovigilance centre with similar methodology found that of 1139 reports, only 1.4% were considered high quality [39]. Furthermore, a study on 999 ADR reports in Brazil noted that only 4.4% were sufficiently completed for causality assessment, whilst the completion rate was not influenced by severity or whether the reaction was well-known [40]. A French study, which assessed completeness based on extended information (e.g. co-morbidities, use of concomitant medications, diagnosis of effects, clinical course, outcome, therapeutic measures, laboratory data and response to de-challenge or re-challenge), in addition to the standard domains, found only 12.7% of the 613 ADR reports submitted by general practitioners to the regional pharmacovigilance centre were ‘well-documented’ [41]. On the other hand, several studies from Sweden, the Netherlands, Norway and India have reported high quality documentation rates of between 48 and nearly 100%, in both community and hospital settings [16, 42–44].

All-in-all, these studies highlighted that quality assessment methods, and hence the quality and completeness of ADR reports, varied widely between centres and settings. The use of differing assessment criteria in these studies also made direct comparison challenging. There were no obvious factors that reliably explained why the quality of documentation varied widely between different centres and geographical regions.

### Risk communication

Risk communication, whereby HCPs communicate regarding an ADR episode in a written/verbal form to patient, family and other health practitioners, is an important aspect of ADR management. The key goal is to provide specific information to eliminate or minimise re-exposure to the offending and structurally related medications [45]. Examples of risk communication include a medication alert card/bracelet for the patient and a concise hospital discharge summary. ADR alerts must also be entered into the patient’s hospital medical records. Where causal medications are still undetermined at the time of patient review in the community or at hospital discharge, and/or where further assessment by a specialist is warranted, an interim advice to avoid suspected medications must also be provided.

To date, risk communication practices by HCPs have not been widely studied.

Teo et al. studied hospitalised patients with cutaneous adverse drug reactions, finding only 1 in 5 discharge summaries included the reaction description [46]. An Australian study of 84 patients with severe cutaneous adverse drug reactions found 13% patients were not given any written information of implicated medications at discharge. Moreover, 26.3% had a mismatch of knowledge regarding what they believed was the implicated medication and what was documented in hospital records [26]. Another multicentre Australian study found that nearly 30% of the anaphylaxis episodes to antimicrobials were not documented in hospital records [28]. A study in the UK showed that, of 127 general practitioners who followed up patients with ADRs after discharge, 89% had no record of patients’ ADRs, having to rely on the patients themselves to provide this information [47].

Ultimately, risk communication of ADRs to patients and colleagues by HCPs remains suboptimal. Risk communication practices thus need to be incorporated in the ADR management processes as a key component to further improve patient safety and quality of care.

## Sources of variation

Many studies have examined the reasons behind under-reporting, yet few studies have evaluated variations in the quality of ADR reports, and risk communication. The latter two factors are more likely influenced by HCPs’ knowledge and skills [48]. Thus, overall clinical competency may significantly contribute to variations in management observed [49].

### Behaviours and attitudes in under-reporting

Reasons for suboptimal reporting rates have been extensively evaluated and can be grouped into several behavioural and attitudinal factors, as outlined in Table 2 [20, 50]. A systematic review of 50 studies by Lopez-Gonzales et al. found that attitudes most frequently associated with under-reporting were ignorance (95%), diffidence (72%), lethargy (77%), indifference and insecurity (67%), complacency (47%) and fear (24%) [20]. Other barriers such as lack of training, infrequent experience with ADRs in practice, unfamiliarity with reporting requirements, lack of time, difficulties locating and completing reporting forms and bureaucratic hurdles were also identified in several studies amongst both undergraduate students and HCPs of various disciplines [51–53]. Reasons for under-reporting amongst physicians included the ADR was already well-known (75.6%), too trivial (71.1%) and uncertain causality (66.3%) [54]. Conversely, ADRs with the highest probability of being reported were serious unknown adverse reactions to a new (81.1%) or to an established medication (72.9%), and serious known reactions to a new medication (65.2%) [54].

**Table 2:** Knowledge and attitudes of healthcare professionals contributing towards under-reporting of adverse drug reactions [20]

1. Complacency – belief that only safe drugs are allowed in the market
2. Fear of possible involvement with litigation or investigation
3. Guilt at having administered treatment that may have harmed a patient
4. Ambition to compile and publish a personal case series
5. Ignorance of the requirements of reporting
6. Diffidence at reporting merely suspected ADRs
7. Indifference to essential role as clinician who should be contributing to medical knowledge
8. Lethargy – a combination of procrastination, lack of interest or time
9. Financial incentives to report
10. Insecurity – that it is nearly impossible to determine whether or not a drug is responsible for a particular ADR

*ADR* adverse drug reaction

### Knowledge gaps in ADR assessment and reporting

Several studies have examined the impact of knowledge gaps, and the need for ADR education as a method of quality improvement.

A systematic review by Reumerman et al. found that most undergraduate medical and pharmacy students felt training was inadequate and their knowledge was insufficient in ADR reporting and pharmacovigilance activities. Prior training in ADR reporting was associated with significantly higher knowledge scores, which correlated with better skills in reporting [55]. Similarly, another systematic review evaluating essential prescribing competencies of final year medical students found that students lacked knowledge of where and how to report ADRs, with little to no training included in undergraduate curricula [56]. Specifically in the Australian context, in a study of community pharmacists, the median score for knowledge of pharmacovigilance systems and reporting requirements was found to be just five out of a possible ten [57]. In the same survey, over 95% believed pharmacovigilance teaching should be included in the undergraduate pharmacy curriculum and almost 90% believed that professional bodies should include this topic in postgraduate professional development activities [57].

Specifically on the knowledge regarding drug hypersensitivity reactions (DHRs), an Australian multicentre study amongst 238 medical doctors and pharmacists surveyed in hospital settings found significant knowledge gaps in the domains of syndromic recognition, causality attribution, antibiotic cross-reactivity patterns and diagnostics/therapy, corresponding to an overall correct score of just 55.6% [48]. Knowledge gaps were similar for key ADR principles assessed, regardless of healthcare profession and seniority level [48]. A lack of medication allergy knowledge in undergraduate medical training was noted in a study from Turkey, which found only 58.5% of questions regarding medication allergy were answered correctly [58].

Collectively, the above studies highlighted that many HCPs received inadequate education and training on several aspects of ADR management and pharmacovigilance in both undergraduate and postgraduate settings, even though these activities form an essential part of HCPs’ professional responsibilities.

## ADR education as quality improvement

The process of ADR assessment and reporting can be complex. Regardless of reporter’s vocation, a comprehensive ADR evaluation requires several technical skills including knowledge and familiarity with medications, their side effect profiles and, to some extent, pharmacokinetic and pharmacodynamic properties [5]. Equally important is having knowledge of ADR patterns/syndromes and pathophysiology. Knowledge of causality attribution based on temporality, awareness of available and validated causality assessment tools, their strengths and limitations and knowledge of cross-reactivity between medication classes are also important [48]. Where causality remains unclear, awareness of available diagnostic tests for further evaluation is helpful. As spontaneous reporting relies heavily on reporters being aware of reportable ADRs, HCPs should have basic knowledge and familiarity with key concepts and requirements. Therefore, dedicated ADR education should be a key strategy to improve the quality of ADR management.

### ADR education curriculum

To standardise pharmacovigilance and ADR teaching within tertiary education framework, the Netherlands Pharmacovigilance Centre, on behalf of the World Health Organization, discussed the development of university curricula at a stakeholders’ meeting in 2016. The meeting identified desired competencies to be taught at the tertiary level, either integrated in existing health science courses or as a stand-alone programme [51]. The proposed curriculum is based on five key aspects: understanding the importance of pharmacovigilance, preventing ADRs, recognising ADRs, managing ADRs and reporting ADRs. It also outlines required knowledge, skills, attitudes and examples of teaching methods to achieve these aims through active learning [51]. Pharmacovigilance and ADR management aspects are incorporated into existing pharmacology education programmes. The curriculum has a healthcare-focused approach, rather than a regulatory focus, to promote patient safety, safe prescribing and reducing medication-related harm.

Complementing these WHO curricular recommendations, Herrera-Comoglio further proposed basic and advanced ADR education contents to be taught in both undergraduate and postgraduate settings (Table 3) [59].

**Table 3:** Topics and content of adverse drug reaction education curriculum [59]

**Topics**	**Content**
Basic ADR principles	Definitions
	Epidemiology
	Population impact
	Classification
	Identification of common ADRs
Knowledge of hypersensitivity reactions	Gell-Coombs classification of underlying pathophysiology
	Clinical syndrome recognition
Pharmacogenetics	Definitions and concepts
	Pharmacogenetics influencing pharmacokinetics and pharmacodynamics mechanisms and pathogenesis of hypersensitivity reactions
	Concept of personalised medicine
Causality assessment	Medication history and timeline
	Clinical indications and differential diagnoses
	Recording and assessment of past ADRs
	Available causality assessment algorithms (e.g. Naranjo, ALDEN, Regi-SCAR, etc.), their strengths and limitations
ADR prevention	Therapeutic decision making
	Knowledge of medication cross-reactivity patterns
	Harm minimisation in prescribing (e.g. dose adjustment, avoidance)
	Effective risk communication to patients and other healthcare professionals
ADR reporting	Reliable sources of information (regulatory information, WHO, LiverTox etc)
	Knowledge of pharmacovigilance systems, reporting requirements and processes
	Compiling a comprehensive high-quality report

*ADR* adverse drug reaction, *ALDEN* Algorithm for Assessment of Drug Causality in Stevens-Johnson Syndrome and Toxic Epidermal Necrolysis, *Regi-SCAR* Registry of Severe Cutaneous Adverse Reaction, *WHO* World Health Organization

### Methods of delivery

Beyond the undergraduate curricula, periodic pharmacovigilance and ADR training should also form a mandatory part of continuing education for HCPs in their respective professional practice [60]. Such education sessions may be conducted by individual health services, universities or professional societies as part of continual professional development programmes. Indeed, the Australian Commission on Safety and Quality in Healthcare (ACSQH) ‘Medication Safety Standard’ stipulates orientation and training of new clinicians/practitioners to the existing ADR management systems and also that periodic competency assessments should also be provided by the healthcare institutions, including hospitals [61]. ACSQH further recommends that training must account for common medication incidents, differing levels of knowledge and experience of HCPs and accessibility to evidence-based resources and guidelines.

Contemporary healthcare education evidence suggests that ad hoc clinical exposures are insufficient to establish clinical competency and that more deliberate repeated practice opportunities achieve a deeper level of mastery [62, 63].

Simulation-based medical education (SBME) permits health professionals to engage in deliberate practice in a safe, learner-centred environment [64]. SBME can be a powerful education method when coupled with directed learning outcomes, structured feedback and competency assessments sequenced at progressive levels of difficulty [64–66]. SBME has been used to establish competency in a range of skills (e.g. decision-making, communication) within a variety of healthcare professions including medicine and pharmacy [64]. Simulated clinician-patient interactive communication sessions for final year medical students centred on medication errors and adverse drug events received very high acceptance rates and improved awareness of knowledge deficits amongst students [67].

In contrast, a literature review of pharmacovigilance education in HCPs suggests that learners prefer deliberate experiential learning using realistic cases rather than simulated scenarios [55]. Other education interventions that can support ADR recognition, management and reporting include problem-based learning and team-based learning especially when incorporated into interprofessional activities amongst HCPs [68]. Whilst there is no definitive answer as to the most effective method of education delivery, the strategies mentioned all rely on the learner’s active retrieval and application of knowledge and skills. This is consistent with evidence suggesting that active learning (e.g. SBME, experiential learning) has a greater impact on knowledge, attitudes and skills than passive learner (e.g. didactic lectures) [69].

### Evidence regarding education interventions and ADR outcomes

Studies have shown that brief education interventions, either stand-alone or in combination with other measures, improve the rate of ADR reporting. A systematic review by Li et al. evaluated multiple strategies in hospital and primary care settings targeted at improving ADR reporting rates [70]. The most common strategies employed were educational sessions, including group presentations or workshops (31.6%) and systemic changes, such as electronic ADR reporting tools (26.3%). Other initiatives included reminders (15.8%), offering an economic incentive (10.5%), utilising telephone interventions (10.5%) and providing feedback to reporters (5.3%). All interventions increased both the rate and number of ADR reports. The point estimate increase in reporting rates was 9.26-fold (95% *CI*: −2.21 to 20.74) for multifaceted approaches and 7.19-fold (95% *CI*: −2.73 to 17.11) for single interventions. Traditional education methods alone increased reporting rates by 4.42-fold (95% *CI*: 0.66–8.19). Electronic reporting tools served as passive facilitators, whilst education sessions and/or reminders proved active promoters of ADR reporting.

A systematic review by Pagotto et al. also found positive impacts in spontaneous reporting practices with education interventions [60]. The studies included in this review adopted multiple interventions, which included distribution of printed educational materials, repeated email reminders, educational lectures, workshops, group dynamic exercises, regular meetings, outreach visits and interviews or questionnaires to reporters [60]. In a cluster randomised controlled trial by Lopez-Gonzalez et al., education was delivered through group sessions and educational materials with particular focus on the importance of ADR reporting, by emphasising ADR–associated morbidity, mortality and cost data, as well as the limitations of clinical trials for pharmacovigilance measures, hence the importance of spontaneous reporting as a post-marketing pharmacovigilance tool. Education intervention improved total ADR reports by 1.65-fold (95% *CI*: 1.08–2.53), serious ADRs by 1.62-fold (95% *CI*: 0.99–2.65), unexpected ADRs by 2.06 (95% *CI*: 1.19–3.55) and high causality ADRs by 1.13 (95% *CI*: 0.72–1.77) over the 8-month follow-up period. However, improvement efforts did not sustain beyond 4 months with a single education session [71].

Education, in the form of outreach visits, has also been shown to improve overall ADR reporting rates (RR 10.23, 95% *CI* 3.81–27.51) and that of serious, high causality, unexpected and new-drug–related ADRs in a cluster randomised controlled trial conducted in Portugal. The outreach visits included presentation of data on ADR–related morbidity and mortality, rate of hospital admission, cost to patients and the health system and information on pharmacovigilance systems. The presentation also addressed the attitudes associated with under-reporting. However, the improvement was also found to be maximal in the first 4 months and waned over time [72].

Although brief educational interventions have been shown to improve ADR reporting rates, pooled results from single forms of education only achieved a modest 2.3-fold increase in rates [42, 71]. In contrast, combining education with reminders, feedback and improved accessibility to reporting resources resulted in a 14-fold increase in reporting [73]. Also, single education sessions rarely provide sustained improvement over time, thus emphasising the importance of repeated reinforcement [71, 72].

Whilst many studies have shown that education strategies can improve overall ADR reporting rates, no studies to date have specifically evaluated the impact of educational interventions on the quality of ADR assessment and reporting, as well as on risk communication and patient safety. Education strategies that focus on the latter quality outcomes are urgently needed.

## Summary and future directions

In hospital settings, spontaneous reporting remains the main method of detecting and monitoring ADRs. Whilst the primary purpose is to contribute toward pharmacovigilance and centralised regulatory activities, the local focus of ADR management should be to promote patient safety through comprehensive assessment, risk communication and safe prescribing.

Hospital ADR management processes vary greatly, from having only individual reporters to a multi-disciplinary ADR review committee, depending on the size of the institution and resources available. Complexity of ADRs encountered also varies, depending on the patient population and specialty services available. Human factors, such as HCP expertise, attitudes and knowledge, affect assessment and reporting practices. Quantitative outcomes, such as the number of ADR reports, can be easily monitored. However, available and practical tools to measure the quality of ADR management remain ill-defined. Certain indicators, such as completeness of ADR reports, which reflect the quality of assessment, and frequency and effectiveness of risk communication practices, need to be monitored for an indication of overall quality of ADR management at an individual healthcare organisation level. Further research is needed to develop validated and easy-to-use tools that can be utilised within hospital environments to measure and quantify quality indicators.

Whilst systemic approaches such as development ADR reporting guidelines, standardisation of reporting forms or electronic reminders/prompts may help improve spontaneous reporting rates, it is still important to address the gaps in knowledge and competency of HCPs in ADR assessment as a quality improvement process. Robust ADR and pharmacovigilance education programmes at both undergraduate and postgraduate levels may bridge some of these identified gaps. Education processes need to be relevant and targeted at appropriate profession and skill levels and reinforced at intermittent intervals for improvements to be sustained. Outcomes of educational interventions should be systematically measured, preferably through quality indicators, and regularly reported.
